# Supplementary material for: Scientia Pharmaceutica, Autorenhinweise 2013
Source: Sci Pharm. 2012 Dec 10;81(1):297–308. [Article in German] doi: 10.3797/scipharm.aut-13-01 (PMC3617667; doi:10.3797/scipharm.aut-13-01)
Supplement: Related texts

# Sub article: Scientia Pharmaceutica, Instructions for Authors 2013

**Published:** 2012-12-10

**Scientia Pharmaceutica** (**www.scipharm.at**), a peer-reviewed journal with quarterly appearance, is published at the end of every quarter of each year. It is a medium for publication of original papers, short communications, a limited number of reviews, and papers concerning pharmaceutical practice, referring to all scientific aspects of pharmacy and related disciplines.

The journal Scientia Pharmaceutica (ISSN 0036-8709, e-ISSN 2218-0532) is currently indexed by Chemical Abstracts (SciFinder), Scopus, PubMed, PubMed Central, Asian Science Citation Index, International Pharmaceutical Abstracts, Google Scholar, Natural Product Updates, CAB Abstracts, EBSCO, Chimica, Medicinal and Aromatic Plants Abstracts, ChemInform, ProQuest, Embase / Excerpta Medica and many others.

Manuscripts should be prepared in English language and should be submitted via e-mail to **office@scipharm.at**. The receipt of the manuscript will be acknowledged via e-mail to the corresponding author.

Only unpublished material will be accepted. If partial results are published they have to be cited correctly and the corresponding publications have to be enclosed. The editors reserve the right to reject papers with linguistic or scientific deficiencies or lacks regarding the preparation of the manuscript (see also criteria for immediate rejection). Prior to the submission of reviews, an abstract must be sent to the editorial office for pre-evaluation.

The submitted manuscripts will be peer reviewed by external reviewers guided by a scientific editor. It is recommended to suggest 3–5 independent and competent reviewers in the cover letter (complete contact information including e-mail is needed). It is incumbent upon the editors to make the final decision.

By submitting a manuscript the submitting / corresponding author agrees that he is authorized by his co-authors to enter into these arrangements and he warrants, on behalf of himself and his co-authors, that:

The article is original, has not been formally published in any other peer-reviewed journal, is not under consideration by any other journal and does not infringe any existing copyright or any other third party rights;

He is/they are the sole author(s) of the article and have full authority to enter into this agreement and in granting rights to Österreichischer Apothkerverlag m. b. H. are not in breach of any other obligation;

The article contains nothing that is unlawful, libelous, or which would, if published, constitute a breach of contract or of confidence or of commitment given to secrecy;

He has/they have taken due care to ensure the integrity of the article. To his/their – and currently accepted scientific – knowledge all statements contained in it purporting to be facts are true and any formula or instruction contained in the article will not, if followed accurately, cause any injury, illness or damage to the user.

The article, if accepted, will be published under the Creative Commons License 3.0 (http://creativecommons.org/licenses/by/3.0/) and is licensed to Österreichischer Apothekerverlag m. b. H. In summary, this means that the copyright stays with the author(s) and that the article is published open access. Hence, anyone will be free to copy, distribute, and display the work, to make derivative works, and to make commercial use of the work under the following conditions: The original author must be given credit (citation) and for any reuse or distribution, it must be made clear to others what the license terms of this work are. Any of these conditions can be waived if the authors give permission.

There are neither author/institution fees nor page charges. Both (online) reading and publishing is free. Information on how to order the print version can be found online: www.scipharm.at/subscription

To achieve a rapid and cost-effective publication as well as a uniform appearance of Scientia Pharmaceutica, authors must adhere to the Instructions for Authors and the electronic manuscripts in Microsoft Word format should be ready for direct reproduction.

## Checklist for Manuscript Submission to Sci Pharm:

□ Latest version of instructions downloaded from www.scipharm.at/authors?
□ Manuscript prepared preferably using the MS Word template?
□ Manuscript spellchecked and (external) language corrections done?
□ One single manuscript file in MS Word format submitted? Figures, schemes, and tables inserted in high resolution into the main text and no landscape format used?
□ Corresponding author designated and e-mail address(es) given?
□ Full names (first, middle and last names) and affiliation of all authors given?
□ Correct style for references? Citations placed in brackets, e.g. [1, 3, 6–9]
□ Criteria for Immediate Rejection read and understood?
□ Cover letter with all relevant information for editor:
  ○Relevant submitted or in press manuscripts
  ○Ethical statements (competing interests, informed consent, human & animal rights)
  ○Complete contact information for independent referees given?

## Guideline for manuscript preparation

It is strongly recommended to look at the style of recent publications in Scientia Pharmaceutica prior to preparing a manuscript. Moreover, a MS Word template should be used and is available for download at http://www.scipharm.at/authors.

### Figures and tables

Figures, schemes, graphs and tables must be inserted in the text appropriately in suitable size and quality. Landscape pages must not be used! Please note that the original size will be reduced to approx. 70% during printing.

Chemical structures should be drawn with a suitable drawing program (preferably ACD/ChemSketch).

### Nomenclature

Chemical nomenclature should follow the rules of the IUPAC and should be proven by professional computer software; botanical nomenclature must be in agreement with the corresponding “International Code” or Englers “Syllabus der Pflanzennamen”.

### Characterization of new compounds / plant extracts

Typically, all new compounds need a detailed analysis with the following state-of-the-art techniques: ^1^H NMR, ^13^C NMR, MS (including fragmentation pattern for EI MS), elemental analysis; MP for solid compounds. Additional techniques such as IR, hrMS, X NMR, 2D NMR, X-Ray, HPLC, etc. may be required and all available data should be given whenever possible. The editors may request the submission of the original spectra.

TLC/HPLC fingerprint for plant extracts and fractions are required.

#### Fictitious Example:

Colorless crystals of mp 118–120 °C (aqu. ethanol). MS (*m/z*, %): 202 (M^+^, 3), 184 (36), 73 (100). IR (KBr), ν, cm ^−1^: 1683, 1645. ^1^H NMR (500 MHz, CDCl_3_, TMS): δ 8.74–8.80 (m, 2H, H-3,5), 8.18 (dd, ^3^*J*_H2,H1_ = 7.7 Hz, ^4^*J*_H2,H7_ = 1.1 Hz, 1H, H2), 7.68 (s, 1H, H-6), … ^13^C NMR (125 MHz, DMSO-*d*_6_, TMS): δ 175.4 (COOH), 136.3 (C-7), 134.1 (C_q_), 128.0 (CH), 127.6 (CH), 54.3 (CH_2_), … HRMS: Calcd. for C_11_H_10_N_2_O_2_: 202.0742. Found: 202.0746. Anal. Calcd. for C_11_H_10_N_2_O_2_: C, 65.34; H, 4.98; N, 13.85. Found: C, 65.04; H, 5.22; N, 13.60.

### Language corrections

It is the responsibility of the authors who publish in a language other than their native one to ensure correct language usage by an external proof reading institution. Papers with poor language will be rejected! In the cover letter the utilized organization and the corresponding job number should be given. Editing certificates such as those from American Journal Experts (20-digit certification code must be provided) are accepted as proof for sufficient language quality of the submitted paper. Exemplary links to some companies that specialize in proof reading of scientific papers can be found at www.scipharm.at/authors. However, it should be noted that Sci Pharm does not recommend a specific organization and does not have any financial relation to one of them.

### Supporting Information

Scans of original spectra, extensive tables and figures can be published as supplementary material in the online version.

### Ethical statements

#### Competing interests

A competing interest exists when an individual (or the individual’s institution) has financial, personal or professional relationships with other persons or institutions that could unduly bias his or her actions or judgments. Competing interests may arise from those relationships, irrespective of whether the judgment is actually affected or only potentially.

e. g. The authors declare no conflict of interest. / ABC holds a patent on … / DEF is a consultant of the company XYZ …

#### Informed Consent & Ethical Approvals (if applicable)

Patients have a right to privacy that should not be infringed without informed consent. Identifying information, including patients’ names, initials, or hospital numbers, should not be published in written descriptions, photographs, and pedigrees unless the information is essential for scientific purposes and the patient (or parent or guardian) gives written informed consent for publication.

Investigations with human subjects must state in the experimental section that the research followed the ethical standards formulated in the Helsinki Declaration of 1964, revised in 2000 (http://www.wma.net/e/policy/b3.htm), and was approved by the institutional human experimentation committee or equivalent. Any doubt about the ethical suitability of the human procedures used will result in the immediate rejection of the paper by the editors.

#### Animal Rights (if applicable)

Investigations with animals must state in the experimental section that the research followed the (inter)national ethical standards for the care and use of laboratory animals and was approved by the institutional animal experimentation committee or equivalent. Any doubt about the ethical suitability of the animal procedures used will result in the immediate rejection of the paper by the editors.

### References

References appearing in the text must be numbered continuously in brackets, e.g. [2–4] and listed in the section “References”. The footnotes or endnotes function of MS Word must not be used! References must be cited similarly to the “Vancouver style” [1] as shown below (books [2], journals [3, 4]). As far as possible, doi-numbers should be given in a separate line.

□ *Author*: In the first line of each journal citation, all authors are given in the order they appear in the original text. The capitalized surname (family or last name) is given first and – after inserting a space – the given (first) names and middle names are converted to initials, for a maximum of two initials following each surname. Separate author names from each other with a comma and a space; end the author information with a period. Insert a soft-return (by pressing both the shift and enter key simultaneously) to start the title line.
□ *Title*: Enter the title of an article as it appears in the original publication. End the title with a period and insert a soft-return to start the journal line.
□ *Journal*: Abbreviate the journal’s name, capitalize all words and abbreviations, do not use periods except after the last word which is followed by a period and a space. Insert the publication year followed by a semicolon and a space. Insert the volume followed by a colon and a space. Do not give issue numbers. Insert both the article’s first and last page, separated by an en-dash (–) without any spaces. End the page number with a period. Insert a soft-return for entering the doi number (if available) or insert a hard-return to continue with a new citation.
□ *Doi-number*: Insert the doi-number without any spaces. End with a hard-return to continue with a new citation. Doi-numbers can be easily found by using the free web-service at http://www.crossref.org/SimpleTextQuery (requires one-time registration; just copy your reference list into the box and press ‘submit’).

It’s the sole responsibility of the submitting authors to provide exactly formatted and correct citations!

## Criteria for immediate rejection

The editors reserve the right to reject submitted papers without peer reviewing, especially in the following cases:

### Language & journal style

Papers with poor language or papers lacking the journal’s formal style as described in these instructions are not acceptable.

### Characterization of compounds

Manuscripts will be rejected if sufficient analytical data (e. g. ^13^C NMR for new compounds or a fingerprint chromatogram for plant extracts) are not provided.

### In-vitro antioxidant activity

Because antioxidant activity is present in all plants, a routine screening with in-vitro assays has little meaning if no clear evidence is given for in-vivo activity.

### Miscellaneous

Routinely determined LD_50_ data for new compounds are ethically not acceptable.

### Missing citations

Papers that do not discuss and cite relevant literature will be rejected.

### Spectrophotometric and electroanalytic methods, Thin-Layer Chromatography

Papers mainly presenting such analyses are typically not acceptable when modern chromatographic methods are available for quantification.

## Simultaneous quantification

Authors have to intensively argue the novelty and the value of their method in comparison to known methods, especially if they add only a new compound to an already well analyzed combination of compounds or if they interchange compounds of already well analyzed combinations (e.g.; A+B+C when A+B, B+C and A+C are already known).

### Non officinal naturals

Typically, papers dealing with the use and development of unapproved natural matrices will be rejected.

### Plagiarism and duplicate publication

Such papers will be rejected and the editors may exclude the authors from future submissions.

## References

[1] International Committee of Medical Journal Editors Uniform requirements for manuscripts submitted to biomedical journals. http://www.icmje.org/.

[2] Hänsel R, Keller K, Rimpler H, Schneider G (1994). Passiflora. Hagers Handbuch der Pharm Praxis.

[3] Katritzky AR, Odens HH, Zhang S (2001). Novel Synthesis of 2,3-Dihydro-1,5-benzothiazepin-4(5H)-ones and 2H-1,4-Benzothiazin-3(4H)-ones. J Org Chem.

[4] Koch C, Reichling J, Schneeleb J, Schnitzler P (2008). Inhibitory effect of essential oils against herpes simplex virus type 2. Phytomedicine.
